# Using X-ray Micro-Computed Tomography to Three-Dimensionally Visualize the Foregut of the Glassy-Winged Sharpshooter (*Homalodisca vitripennis*)

**DOI:** 10.3390/insects13080710

**Published:** 2022-08-07

**Authors:** Nabil Killiny, Craig R. Brodersen

**Affiliations:** 1 Citrus Research and Education Center, IFAS, University of Florida, 700 Experiment Station Road, Lake Alfred, FL 33850, USA; 2 School of the Forestry and Environmental Studies, Yale University, 195 Prospect, New Haven, CT 06511, USA

**Keywords:** *Homalodisca vitripennis*, sharpshooter, foregut, precibarium, cibarium, *Xylella fastidiosa*, vector, microCT scan

## Abstract

**Simple Summary:**

The foregut of the glassy-winged sharpshooter is difficult to dissect and observe in three dimensions with SEM or TEM. We describe an X-ray micro-computed tomography (microCT)-based method for studying the GWSS foregut including the precibarium and the cibarium, with high resolution. MicroCT imaging showed great promise for the study of the tissues and structures of small insects.

**Abstract:**

Dissecting the heads of small insects belonging to the order Hemiptera for detailed anatomical investigation with light or scanning electron microscopy is difficult, time-consuming, and destructive, often resulting in sample preparation artifacts. Nevertheless, the structural details of these insects often hold critical information regarding their interactions with bacterial pathogens. For example, the glassy-winged sharpshooter (GWSS) is an efficient vector of the bacterium *Xylella fastidiosa*, the causal agent of Pierce’s disease in grape vines, but the foregut of this species is exceptionally difficult to dissect. Here, we describe a simple, non-destructive method to investigate the structure of the anterior gut of GWSS using high-resolution micro-computed X-ray tomography (microCT). The use of microCT eliminates the need for destructive dissection and reveals the morphology of small insects in three dimensions, allowing the user to virtually dissect the sample. The use of microCT imaging is a promising and powerful tool in the entomological sciences for studying the structures of vector insects, especially for difficult-to-dissect regions such as the foregut.

## 1. Introduction

Sharpshooter leafhopper insects (Hemiptera: Cicadellidae) feed on the xylem sap of a range of host plant species. Some sharpshooter species transmit *Xylella fastidiosa* (Xf) into the plant during feeding on xylem sap [1,2]. Within the chitinous mouthparts, specifically, the foregut, bacteria form a regular biofilm [3], which facilitates adhesion to the foregut and helps extract nutrients from the host’s alimentary canal [4]. The transmission of these bacteria by the insect to the host plant can lead to many diseases, including Pierce’s disease of grapevine (PD) and citrus variegated chlorosis (CVC). Broadly, these diseases are responsible for significant economic losses due to decreased yield and additional maintenance costs for growers. Despite the economic importance of this plant–vector–pathogen association, our understanding of the fundamental biology of the interactions between Xf and one of the primary vectors, the glassy-winged sharpshooter (GWSS, Homalodisca vitripennis (Germar)), is not well-studied.

The obstacles to our study of the distribution of Xf within the foregut of the GWSS are the scale, internal complexity, and fragility of these structures, along with their physical location deep within the head. Thus, visualizing the foregut and the sites of biofilm accumulation along the pathway through the mouthparts of these insects presents a major technical challenge. Traditionally, the foregut and mouthparts have been visualized with either scanning electron microscopy (SEM) to observe foregut surface structures and transmission electron microscopy (TEM) to determine the interactions between the biofilm and the chitinous surfaces within the insect [2]. However, TEM requires lengthy embedding and sectioning and provides two-dimensional images that must be serially photographed and then reconstructed into a composite image [5]. While SEM provides excellent images of surface features and provides some depth of field, the many complex internal structures can be easily damaged while samples are dissected to expose the correct surfaces with an appropriate viewing angle. Furthermore, both SEM and TEM are destructive, which inherently limits the visualization of a given sample to a single opportunity, with known uncertainties and artifacts associated with the extensive sample preparations that are time-consuming, technically challenging, expensive, and often require hazardous chemicals.

X-ray computed tomography (CT) imaging has been in wide use for medical imaging for decades and has recently been developed as a non-destructive diagnostic imaging tool for a wide range of biological samples. Based on the same principles as those of medical X-ray computed tomography imaging systems, microCT is useful for both soft and mineralized tissues, with minimal sample preparation required to produce three-dimensional (3D) images with excellent contrast [6]. With this method, hundreds to thousands of two-dimensional X-ray projection images are taken as the sample rotates around 180°, which are then reconstructed into virtual 3D image stacks by computer software. In non-human research, the technology has been applied to the fields of invertebrate and vertebrate zoology, embryology, taxonomy, comparative biology, the plant sciences, and many other fields. The use of microCT has recently been applied to entomology as a means of non-destructively analyzing the internal anatomy of insects [7,8,9,10,11,12]. With the most recent developments in microCT imaging, either at large-scale synchrotron facilities or lab-based systems, the resulting images provide high-resolution, 3D visualizations, detailed measurements, surface morphology, and virtual cross-sections of internal structures in a non-destructive manner [13].

One of the primary benefits of microCT technology is that specimens can be viewed in a “wet state” and need not be translucent or fluorescent, as required by other imaging methods, and whole organisms can be analyzed with submicrometer resolution without dissection or extensive histological preparation [8]. One of the earliest uses of microCT to investigate insects was to examine the specimens of the family Cupedidae, some of the most primitive members of the beetle order Coleoptera. Comparing images obtained from SEM and microCT, SEM provided detailed external structures such as scales and sensillae, which covered the features revealed only by microCT, such as the posterior tentorial pit [7]. Based on the results using this technology, the morphology, function, and phylogeny of an extant species of Archostemata were determined. Hörnschemeyer et al. [7] noted that X-ray technology was especially useful in visualizing the head musculature, organs, and other structures that were not deformed due to the sectioning or cutting of the specimen, and sample preparation was reduced from two weeks to three days. Furthermore, specimens were still intact and available for further study. The disadvantages at that time were lower resolution (which is now greatly improved), and similarity in X-ray absorption between neighboring structures, resulting in a lack of contrast [7].

In recent years, increased resolution, phase contrast, and overall instrument sensitivity toward differences in tissue density have been improved [8,14]. In fact, millimeter-sized living organisms can now be visualized while performing actions such as breathing and feeding to determine the structures and functions of soft tissues, such as nerve and muscle insertions, which are important for biomechanical analyses [14]. Additionally, the microCT technique was used to investigate soft tissues in small insects. MicroCT was used to visualize the vestigial alimentary canal in adult oestrid flies after being stained with 0.5 M iodine [15]. Adult oestrid flies showed a reduced alimentary canal, while the cardia and the diameter of the digestive tract decreased, and the helicoidal portions of the midgut, crop, and salivary glands were absent. This finding indicated that microCT can be a useful technique in insects’ internal morphology.

Here, we used microCT imaging to specifically target the sites of bacterial adhesion and biofilm formation in the foregut of the GWSS to better characterize internal structures and develop a framework for studying the vector–pathogen interaction. Three sites have been identified for bacterial adhesion in the foregut: the precibarium and food meatus, inside the cibarium chamber, and the longitudinal and diaphragm/apodemal grooves [4]. Based on these findings, we examined the mouthparts and foregut structures using synchrotron microCT imaging to virtually dissect the foregut to determine the practicality of using this technology for future experimental applications.

## 2. Materials and Methods

### 2.1. Insects

A laboratory population of the glassy-winged sharpshooter, *Homalodisca vitripennis*, was initiated with field-collected insects from Fillmore, CA, in 2009. Multiple tent-shaped, 60 cm × 60 cm × 60 cm bug dorms (BioQuip, Rancho Dominquez, CA, USA) were set up, each seeded with 90–120 male and female adults. Four to six large basil (*Ocimum basilicum*, Lamiaceae) plants were placed in each of the bug dorms to provide ample feeding. Plants were replaced every 2–3 weeks. Only adult sharpshooters were used throughout this study. All the greenhouse populations were maintained for 3–4 generations before the experiments and were free of *X. fastidiosa* before the experiments began, as it is not transovarially transmitted [16]. Collected insects were treated with CO_2_ to paralyze them prior to head dissection using forceps. Dissected heads were preserved in absolute ethanol prior to microCT imaging.

### 2.2. High-Resolution X-ray Micro-Computed Tomography Imaging (MicroCT)

To investigate the structure of mouthparts of *H. vitripennis*, we scanned the dissected heads with microCT imaging, a non-invasive imaging tool based on X-ray absorption. Scanning was performed at the Lawrence Berkeley National Laboratory Advanced Light Source, beamline 8.3.2, following the methods of Brodersen et al. [17].

Individual sharpshooter heads were allowed to air-dry for approximately 24 h to evaporate all the ethanol preservatives and were then loaded into a polyamide tube and mounted in the microCT system. The heads were imaged at 24 keV with the collection of 1024 2D projection images over 180° in continuous tomography mode at two magnifications, resulting in a 3.25 µm and 0.65 µm voxel resolution. The raw tomographic images were reconstructed with the Octopus software (University of Ghent, Ghent, Kortrijk, Belgium) and visualized with the Avizo software (VSG Inc., Burlington, MA, USA) using a Lenovo desktop computer with 128GB of RAM.

## 3. Results and Discussion

**Figure 1:**
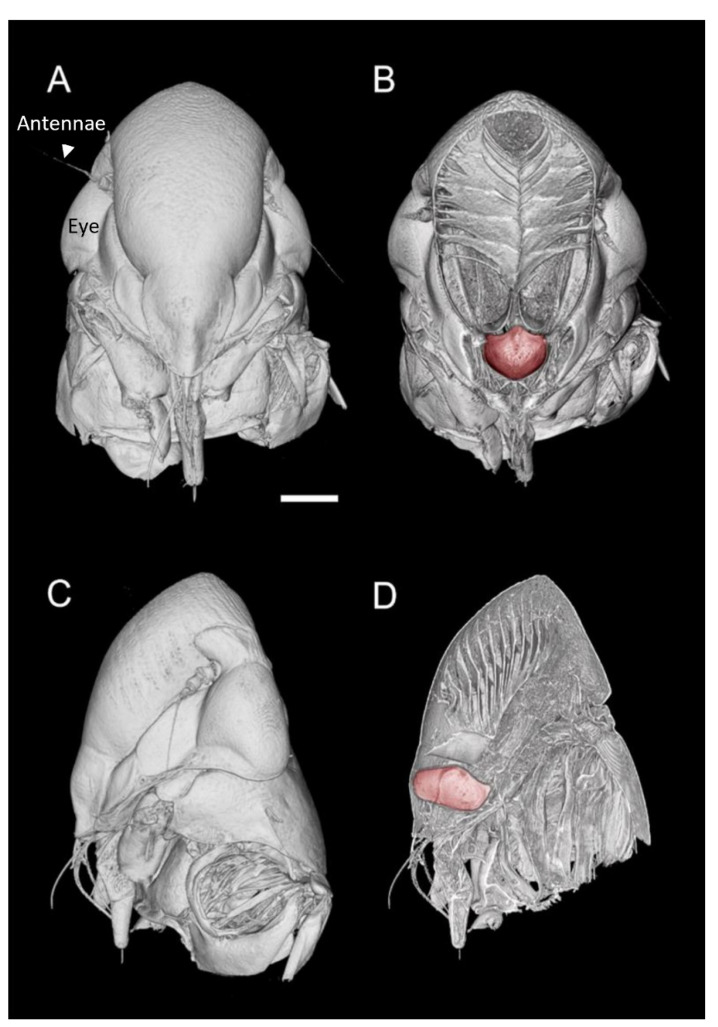
MicroCT three-dimensional volume renderings of the GWSS head at low magnification presented in two orthogonal views (**A**,**C**) and paired with renderings where a virtual slice bisects the head to expose the mouthparts and foregut (false-colored in red) (**B**,**D**). Bar = 250 µm.

**Figure 2:**
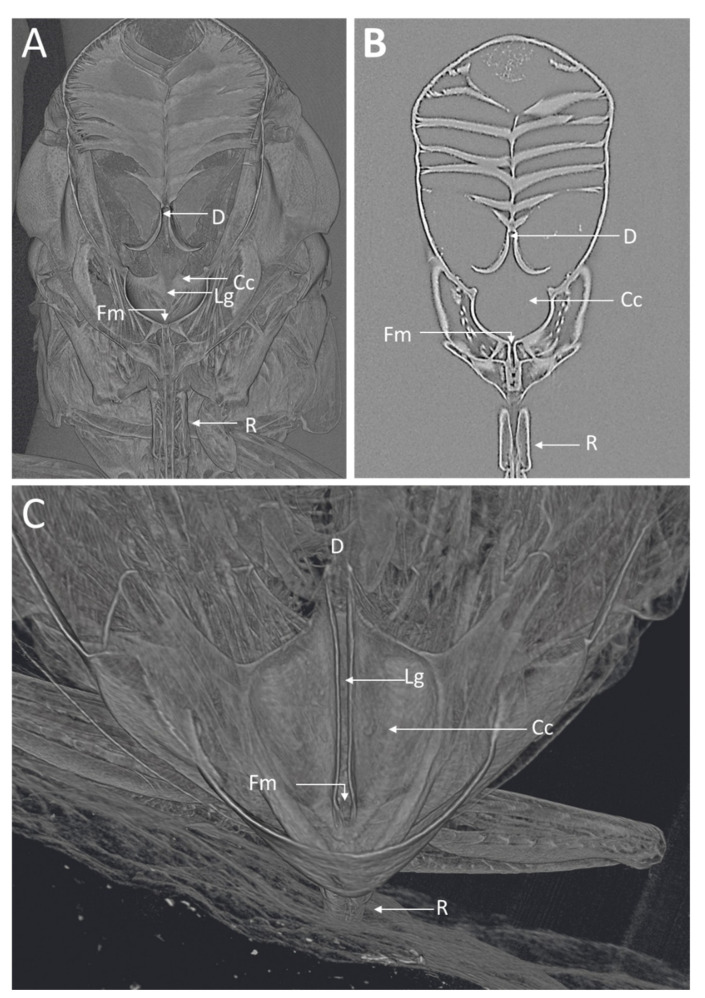
MicroCT images of the GWSS head virtually dissected to expose the foregut and cibarium region. The 3D (**A**) and 2D (**B**) microCT images clearly show various structural details where *Xylella fastidiosa* is known to accumulate and form biofilms. (**C**) 3D microCT volume rendering for a close-up of the cibarium area. D, diaphragm; Lg, longitudinal groove; Cc, cibarium chamber; Fm, food meatus; R, rostrum.

**Figure 3:**
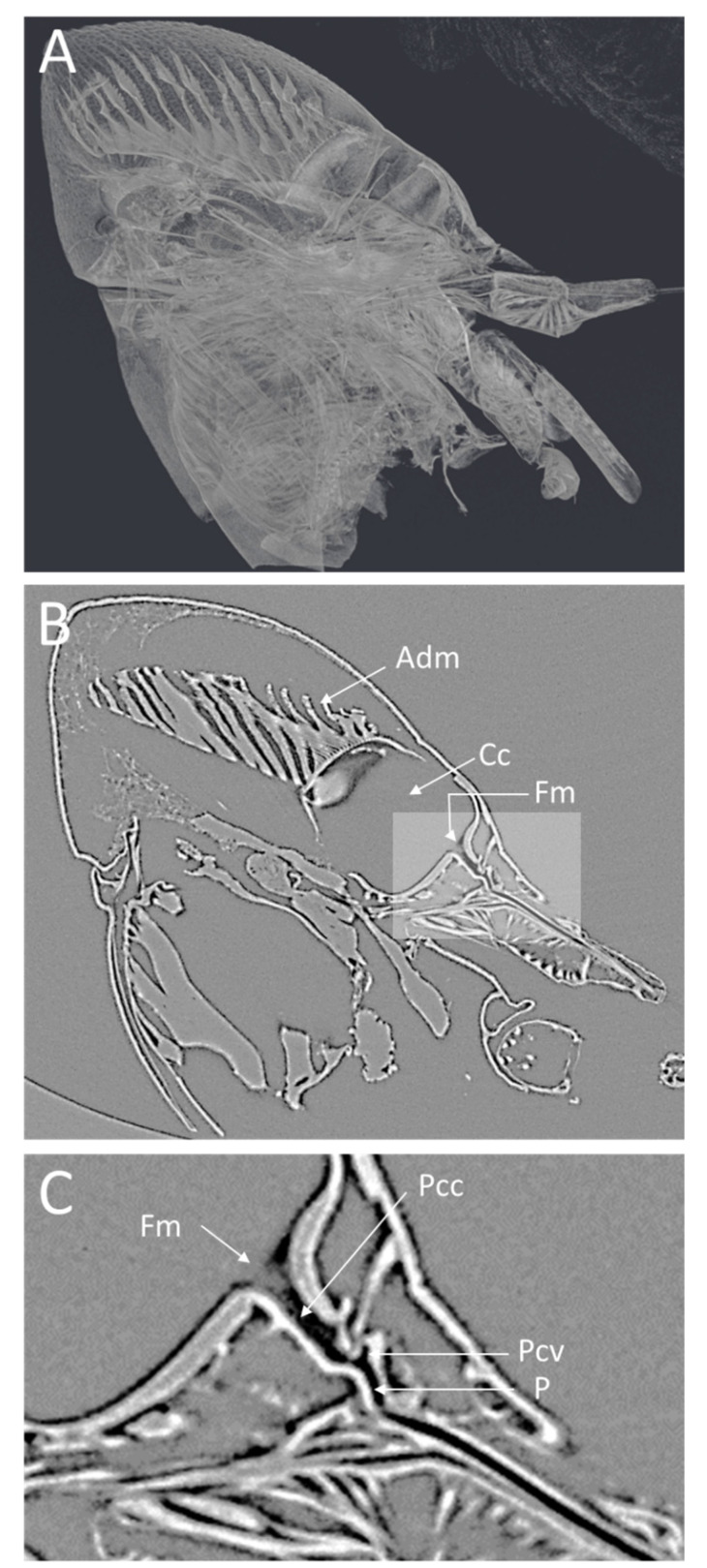
Low-magnification microCT images showing a medial virtual cross-section through the GWSS head. In (**A**), the semitransparent 3D volume rendering shows the whole head and internal structures and musculature. The 2D medial cross-section in (**B**) shows a single virtual slice positioned to bisect the cibarium chamber and the mouthparts where *Xylella fastidiosa* forms biofilms. A higher magnification view of the foregut (**C**) shows the precibarium, precibarium valve, and other features. Adm, apodeme of dilator muscle; Cc, cibarium chamber; Fm, food meatus; Pcc, precibarium canal; Pcv, precibarium valve; P, precibarial valve’s pit.

**Figure 4:**
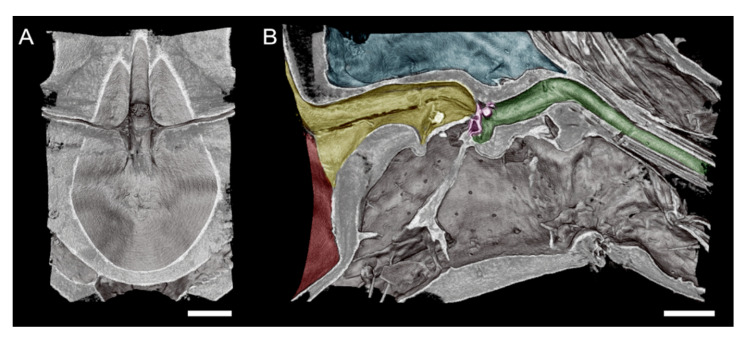
High-magnification microCT images of a subvolume of the GWSS head targeting the cibarium and foregut where *Xylella fastidiosa* is known to form biofilms. A superior horizontal section exposing the cibarium chamber (**A**). An orthogonal view of the same subvolume of the GWSS head reveals the complexity of the pathway through the foregut and mouthparts (**B**). Red shaded area in shows the cibarium chamber, and yellow area shows precibarium). The precibarium valve (purple) is also visible with microCT imaging at this magnification, as well as the precibarial value’s pit (green). Bars = 50 µm.

The microCT imaging of the GWSS heads revealed that the internal structures of these insects, and in particular the mouthparts including the foregut, cibarium, and precibarium, could be visualized with this method. Visualizations performed at the lower magnification were sufficient for observing the gross morphology of the entire head but were insufficient for making detailed observations and measurements of the structures within the foregut (Figure 1 and Figure 2). Increasing the magnification decreased the field of view but allowed for more detail at the 0.65 µm voxel resolution (Figure 3 and Figure 4).

Visualizations with this method allowed for both two- and three-dimensional investigations of the internal structures (Figure 1, Figure 2, Figure 3 and Figure 4), as well as virtual dissections of the samples from any plane or orientation (Video S1). The chitinous structures were easily visible with excellent contrast with essentially no sample preparation (Figure 1B) other than initial ethanol fixation and subsequent air-drying prior to imaging. The images of the virtually dissected head (front view; Figure 2A) show the distinct parts of the cibarium area including the cibarium chamber, diaphragm, food meatus, longitudinal groove, and rostrum.

The lateral view of the GWSS head (Figure 3A) also shows the distinct parts of the cibarium area. The microCT (Figure 3B) shows a clear view of the apodeme of the dilator muscle, cibarium chamber, and food meatus. The close-up view of the cibarium area clearly shows the precibarium canal, precibarium valve, and precibarium valve’s pit (Figure 3C).

The head view in (Figure 3A) shows the hypopharynx, epipharynx, food meatus, cibarium chamber, and the longitudinal groove. The lateral view clearly shows the distinct parts of cibarium area, food meatus, precibarium canal, precibarium valve, precibarium valve muscle, and precibarium valve’s pit (Figure 4B). The sequential sectioning of the GWSS head shows an example of how the head can be rapidly dissected to reveal the cibarium and precibarial area at high magnification (Video S2).

There are many challenges to studying the anatomy of small insects such as those in the order of Hemiptera, which include important agricultural pests such as aphids, psyllids, and leafhoppers. Traditionally, their study requires expert dissection under a stereomicroscope followed by scanning and/or transmission electron microscopy (SEM, TEM). After dissecting, embedding, and sectioning, the fragile chitinous material is difficult to section for TEM, and getting the structures properly positioned for SEM is time-consuming and often fails. Sputter-coating the samples for SEM observation also has the potential to obscure the detailed surface structures. MicroCT is advantageous because it easily visualizes the external and internal anatomy, producing high-definition, dynamic images unavailable by other methods, and with minimal sample preparation. Furthermore, the ability to virtually dissect the sample and easily change the orientation of the exposed surface allows the user to quickly visualize multiple parts of the same sample.

One disadvantage of microCT is that it often does not work well with soft tissues without specific contrast staining, but in the case of insects, the chitin strongly absorbs X-rays, leading to excellent contrast. Thus, studying the body cavities such as the head, including the cibarium, precibarium, and mouthparts is possible, with no staining or complicated sample preparation.

Our microCT images are of comparable quality to static SEM images from previous studies [2,4,18,19] but with the flexibility to repeatedly section the sample to expose a region of interest as many times as desired. In a recent study on the meadow spittlebug, *Philaenus spumarius*, using light and electron microscopy, the morphology and the ultrastructure of the precibarial valve were illustrated [20]. The images presented in Figure 3 are comparable in magnification to SEM images but can be performed in 3D to specifically target anatomical features that are challenging to visualize with traditional methods (Figure 4, Video S2). Using microCT, we were also able to identify the precibarium valve and precibarium valve’s pit, which has also been described by previous reports using scanning electron microscopy [2,19]. The cibarium valve plays a significant role in GWSS feeding. In fact, the flow direction of the ingested sap is controlled by two valves in the GWSS: the cibarium valve and the cardiac (esophageal) valve [2]. Interestingly, the fluid dynamics in the functional foregut of two *X. fastidiosa* vectors were recently studied [21]. This method could, therefore, be used to study the GWSS with and without Xf and possibly in live insects given the fast-scanning times that are possible at synchrotron facilities (<15 min per scan).

Our simple sample preparation protocol and imaging methodology using synchrotron-based microCT show great promise for the use of this technology for studying tissues and anatomical structures that are often difficult to visualize with traditional methods. Pairing microCT with SEM, TEM, light microscopy, or confocal imaging also has the potential to verify the presence or absence of imaging artifacts associated with certain methods. This verification will be an important future step, as synchrotron microCT imaging is not widely available; however, lab-based systems are increasingly common at major research institutions and provide an alternative. Future work should compare synchrotron and laboratory source microCT with traditional imaging methods for use in entomological applications.

## Data Availability

All data are available in this paper.

## Supplementary Materials

The following supporting information can be downloaded at: https://www.mdpi.com/article/10.3390/insects13080710/s1, Video S1: Virtual dissections of the samples from any plane or orientation title. Video S2: A sequential sectioning of the GWSS head shows an example of how the head can be rapidly dissected to reveal the cibarium and precibarial area at high magnification.
